# A Short-Term Comparative Evaluation of Multiple Treatment Modalities for Meibomian Gland Dysfunction: A Prospective Clinical Study †

**DOI:** 10.3390/healthcare13161992

**Published:** 2025-08-14

**Authors:** Mübeccel Bulut, Ali Hakim Reyhan, Gökhan Yüzbaşı

**Affiliations:** 1 Necip Fazıl City Hospital, 46050 Kahramanmaraş, Turkey; 2 Ophthalmology Department, Faculty of Medicine, Harran University, 63300 Şanlıurfa, Turkey; alihakimreyhan@gmail.com; 3 Uğur Eye Hospital, 46040 Kahramanmaraş, Turkey; gokhan_yuzbasi@hotmail.com

**Keywords:** antibiotics, artificial tears, intense pulsed light therapy, meibomian gland dysfunction

## Abstract

**Purpose:** The aim of this study was to evaluate the different approaches used in the treatment of meibomian gland dysfunction (MGD). **Materials and Methods:** This open-label, single-center, prospective pilot study with a parallel-group design was conducted in February 2025. Ninety-two patients presenting to our clinic with symptoms and signs of MGD were enrolled and diagnosed according to Japanese MGD diagnostic criteria. Patients were assigned to five treatment groups: conservative management alone, conservative management plus intense pulsed light (IPL) therapy, conservative management plus oral azithromycin, conservative management plus oral doxycycline, and conservative management plus topical cyclosporine. Conservative management consisted of preservative-free artificial tears containing polyvinyl alcohol and povidone, warm compresses, and eyelid hygiene. Primary outcome measures included ocular surface parameters such as the Ocular Surface Disease Index (OSDI), tear break-up time (TBUT), Standard Patient Evaluation of Eye Dryness (SPEED) scores, and meibomian gland parameters evaluated using a slit-lamp examination. All parameters were assessed at baseline and during follow-up examinations after treatment initiation to observe changes in symptoms and signs. **Results:** A statistically significant increase was determined in meibum grade plugging (grades 0–3; higher = greater obstruction) and Marx line scores with IPL therapy (*p* < 0.05). The group receiving doxycycline treatment exhibited a significant improvement in OSDI and SPEED scores, plugging, TBUT, and meibum grades. The group receiving cyclosporine registered a significant improvement in OSDI and SPEED scores, plugging, TBUT, and meibum grades. A statistically significant increase was observed in the conservative treatment group in terms of lid margin irregularity, Marx line score, and OSDI and SPEED scores. In the group receiving azithromycin treatment, plugging, lid margin irregularity, TBUT, and OSDI and SPEED scores increased significantly. Compared with conservative care, the doxycycline group exhibited lengthened TBUT and lowered meibomian gland plugging and symptoms (*p* < 0.05), and the oral azithromycin group achieved a similar TBUT gain with slightly greater symptom relief. The topical cyclosporine group principally registered improved lid vascularity and meibum quality (*p* < 0.05), while the IPL group achieved the greatest overall improvements, reducing plugging and Marx line scores and adding ≈3 s to TBUT (*p* < 0.05 for all endpoints). Doxycycline exhibited notable short-term improvements, with 35.26% meibum grade improvement and a 40.48% foaming response, while IPL therapy demonstrated substantial OSDI improvements at 54.06%, with traditional parameters indicating limited treatment responsiveness. **Conclusions:** Various methods can be used in the treatment of MGD. All the conservative treatment methods used in this study were successful.

## 1. Introduction

Meibomian gland dysfunction (MGD) constitutes the primary etiology of dry eye disease in clinical practice [1]. This condition, characterized as an evaporative form of dry eye, results from terminal duct obstruction of the meibomian glands, leading to alterations in the lipid layer and lid margin integrity. Patients with MGD experience diminished meibum secretion with increased viscosity and lid margin keratinization. The consequent tear film instability compromises ocular surface wetting, precipitating evaporative dry eye syndrome and triggering inflammatory processes that further exacerbate patient discomfort and visual disturbance [2,3]. Understanding these pathophysiological mechanisms is essential for the development of targeted therapeutic interventions capable of addressing both the mechanical obstruction and inflammatory components of this highly prevalent condition.

Changes in meibum secretion can lead to increased colonization of micro-organisms along the lid margin, which may in turn contribute to infections [4]. The initial approach to treatment usually involves conservative measures such as warm compresses, eyelid massage, and maintaining eyelid hygiene with cleansers that include tea extract combined with the use of artificial tears [5,6]. However, whether the underlying cause of MGD represents an infection at the lid margin or if the infection develops as a consequence of MGD remains controversial [7].

Antibiotics play an important role in the treatment of both blepharitis and MGD, because bacteria produce pro-inflammatory substances capable of exacerbating the condition [8]. Tetracycline antibiotics (such as minocycline and doxycycline) are frequently used, since these not only inhibit bacterial growth but also reduce inflammation by suppressing bacterial lipase activity and controlling pro-inflammatory mediator production [9,10]. Azithromycin, a macrolide antibiotic, functions in a similar way by limiting bacterial growth, reducing the release of lipase, and modulating pro-inflammatory molecules. It also helps to regulate the function and secretion of the meibomian glands. Azithromycin can be administered both topically and orally [11,12].

Intense pulsed light (IPL) therapy represents another treatment modality. This utilizes a high-intensity, non-coherent light source (with wavelengths ranging from 500 to 1200 nm) that stimulates collagen production, targets and destroys abnormal blood vessels, and helps reduce the viscosity of meibum through heat. IPL has also been shown to be effective against Demodex mites [13]. A further option is cyclosporine A, available as a 0.05% topical emulsion, which is a calcineurin inhibitor and the first drug approved by the Federal Drug Administration for treating dry eye disease [6].

The primary aim of this descriptive pilot study was to characterize the short-term clinical evolution and treatment response patterns of five therapeutic approaches toward obstructive MGD, and to assess their feasibility for future larger-scale randomized controlled trials. Secondary objectives included evaluating changes in patient-reported symptoms using validated questionnaires and conducting exploratory statistical comparisons to inform the design of future comparative studies.

## 2. Materials and Methods

This open-label, single-center, prospective pilot study with a parallel-group design was conducted in February 2025 at the Kahramanmaraş Necip Fazıl City Hospital Ophthalmology Department in Türkiye. This study employed a non-randomized, parallel-group design comparing five distinct treatment approaches toward MGD, with demographic matching for age and gender distributions across treatment groups to ensure baseline comparability. Once the study plan had been prepared, patients who presented to the polyclinic with irritative eye complaints within the specified time period, who were diagnosed with MGD as a result of a detailed ophthalmological examination, and who did not meet the exclusion criteria were included.

Ninety-two consecutive patients with clinically confirmed MGD were enrolled during the study period, with no socio-demographic exclusion criteria being applied. Allocation to the five treatment arms followed a structured yet pragmatic algorithm that integrated objective clinical findings and documented contraindications, patient preference after standardized counseling, and practical considerations such as drug or device availability and scheduling constraints. Each participant was thus matched to the most appropriate adjunctive modality while maintaining comparable age and sex distributions across the groups, thereby minimizing selection bias and preserving inter-group comparability. All study procedures complied with the principles of the Declaration of Helsinki, and approval was granted by the Harran University ethics committee (decision no. HRÜ/25.03.33). Written informed consent was obtained from all participants prior to enrollment.

All patients underwent a comprehensive ophthalmological assessment, comprising a slit-lamp biomicroscopic examination, a detailed evaluation of anterior segment structures and a fundoscopic examination, the determination of best-corrected visual acuity, and measurement of intraocular pressure using applanation tonometry.

Clinical examinations, including slit-lamp biomicroscopy, TBUT measurements, lid margin assessments, meibum expressibility testing, and grading of MGD parameters, were conducted by two experienced ophthalmologists (M.B. and G.Y.), both specializing in ocular surface disorders. Standardized examination protocols were employed to ensure consistency across all assessments, and, in order to minimize inter-observer variability, all examining ophthalmologists underwent standardization training sessions prior to study initiation. Calibration exercises were performed using standardized cases, and assessment protocols were reviewed to ensure consistency in grading scales and measurement techniques.

### 2.1. Inclusion Criteria

Obstructive MGD was diagnosed based on the Japanese MGD diagnostic criteria [14]. The diagnosis was confirmed by the presence of ocular symptoms together with abnormal findings in the meibomian gland orifices, such as increased vascularity or the detection of meibomian gland orifice obstruction, as evidenced by plugging and reduced meibum secretion with the application of moderate digital pressure.

### 2.2. Exclusion Criteria

Exclusion criteria included signs of allergic or infectious conjunctivitis, ectatic corneal conditions, a history of prior ophthalmic surgery, current contact lens use, and the receipt of any eye treatment within the previous three months. Refractive error exclusions included high refractive errors (spherical equivalent > ±6.00 diopters) to minimize potential confounding effects on tear film stability and ocular surface measurements. Patients with systemic diseases known to significantly affect tear film production and ocular surface health, including diabetes mellitus, autoimmune rheumatological diseases (Sjögren’s syndrome, rheumatoid arthritis, and systemic lupus erythematosus), and thyroid disorders (both hyperthyroidism and hypothyroidism) were excluded from this study.

### 2.3. Patient Group Allocation

Patients were systematically allocated to the various treatment groups using a multifactorial approach. Clinical assessment parameters, including MGD severity, previous treatment history, and ocular comorbidities, were evaluated for each case. Following comprehensive patient counseling regarding all available treatment modalities, associated risks, and expected benefits, patient preferences were incorporated into the allocation process. Medical contraindications were carefully assessed, with patients with contraindications to oral antibiotics being excluded from the doxycycline and azithromycin treatment groups, while those with a known hypersensitivity to cyclosporine were excluded from topical cyclosporine therapy. Treatment accessibility factors, including IPL device availability and patient scheduling constraints, were considered during allocation. Demographic matching was prioritized to maintain comparable age and gender distributions across all treatment groups. Treatment allocation was performed by the treating physicians using a structured decision-making protocol based on clinical severity assessments using Japanese MGD criteria, patient-specific contraindications, medical history, patient treatment preferences, and practical considerations, including cost and accessibility. To maintain inter-group comparability, patients were stratified by age and gender during enrollment, with baseline clinical parameters balanced across groups. This approach enabled the systematic evaluation of real-world treatment patterns while preserving methodological rigor through standardized outcome measures and predetermined assessment timepoints.

Following a comprehensive ophthalmological evaluation, standardized assessments were conducted, including Ocular Surface Disease Index (OSDI) and Standard Patient Evaluation for Eye Dryness (SPEED) scores. The tear break-up time (TBUT) test was applied, and detailed lid margin evaluations were performed to assess ocular surface integrity. All patients were subsequently recalled for a one-month follow-up examination, during which the initial assessment protocols were repeated to monitor clinical response patterns and treatment evolution.

### 2.4. OSDI and SPEED

The OSDI and SPEED tests, applied at the first examination and during follow-ups, investigate patients’ dry eye symptoms.

The 12-item OSDI and the 4-item SPEED questionnaire were administered to all participants to yield a quantitative assessment of the impact of dry eye on quality of life (see Supplementary Tables S1 and S2).

Improvements in these scores were also measured in proportion to improvements in MGD treatment [15,16,17,18].

### 2.5. TBUT

Tear film stability was evaluated using the standardized TBUT assessment. Following the instillation of 1% fluorescein dye into the conjunctival sac, the time interval between the last complete blink and the first appearance of a corneal black spot in the stained tear film was measured under cobalt blue illumination. In order to ensure reliability, three consecutive measurements were obtained for each subject, and the mean value was calculated and recorded as the definitive TBUT value.

### 2.6. Grading of Lid Margin Findings and Meibum Expressibility

Lid margin findings were assessed at the slit-lamp examination using standardized grading scales. Telangiectasia was evaluated on a scale of 0–3, with 0 indicating no findings, 1 indicating mild telangiectasia, 2 indicating moderate telangiectasia, and 3 indicating severe telangiectasia. The mucocutaneous junction was similarly graded on a scale of 0–3.

In order to determine Marx line scores, 1% fluorescein drops were instilled on the lower eyelid, and the patient was instructed to blink several times. The lower eyelid was then partitioned into three regions—outer, middle, and inner—and scores were assigned based on the extent of fluorescein contact with the meibomian orifices (MOs). Specifically, a Marx line score of 0 was assigned when the line coursed on the skin side of the MO line without contacting the orifices, 1 when portions of the Marx line touched the MOs, 2 when the Marx line passed directly through the MOs, and 3 when the Marx line coursed along the eyelid margin side of the MOs. Lid irregularity, plugging, and foaming were each graded on a scale of 0–2, on which 0 signified no findings, 1 signified mild findings, and 2 signified severe findings [14,19].

The expressibility of meibum from the central area of both the upper and lower eyelids was evaluated semi-quantitatively using a scale of 0–3. A score of 0 indicated that clear meibum was readily expressed, 1 that cloudy meibum was expressed with mild pressure, 2 that cloudy meibum required more than moderate pressure for expression, and 3 that meibum could not be expressed even under strong pressure [20,21] (Table 1).

### 2.7. IPL

An Eye-light^®^ device (Topcon, Bologna, Italy, EYC5FA29I41321K) was employed for the IPL procedure. Initially, 0.5% proparacaine anesthetic eye drops were instilled in both eyes. The treatment involved administering a series of 20 overlapping light pulses to the targeted periorbital skin, extending from the right temple across the lower eyelid, including the nasal bridge, and continuing to the left temple along the lower eyelid. A 590 nm filter in combination with a 6 mm cylindrical light guide was employed on the handpiece [22,23].

### 2.8. MGD Patient Treatment Groups

Patients diagnosed with MGD were stratified into five distinct groups based on the various treatment modalities, all of which were applied for one month.

#### 2.8.1. Group 1 (Conservative Treatment Only)

The patients in Group 1 received conservative management only. All members were prescribed preservative-free artificial tears (four times daily) containing polyvinyl alcohol and povidone (Novaqua, DEVA Holding Inc., İstanbul, Türkiye). A comprehensive conservative regimen was recommended, which included the application of warm compresses, eyelid massage prior to bedtime, and cleansing the eyelashes with an eyelash shampoo.

#### 2.8.2. Group 2 (Conservative Management with Adjunctive Oral Doxycycline Therapy)

The patients in Group 2 were administered oral doxycycline 100 mg (Monodox, DEVA Holding Inc., İstanbul, Türkiye) as an adjunct to the conservative management regimen. The dosage was structured with twice-daily administration during the initial week, and once daily for the subsequent three weeks, for a total treatment duration of one month.

#### 2.8.3. Group 3 (Conservative Management with Adjunctive Oral Azithromycin Therapy)

In Group 3, oral azithromycin 500 mg (Azitro, DEVA Holding Inc., İstanbul, Türkiye, 500 mg) was administered as a single daily dose for three consecutive days in conjunction with the conservative treatment regimen.

#### 2.8.4. Group 4 (Conservative Management with Adjunctive Topical Cyclosporine Treatment)

In Group 4, a topical cyclosporine 0.05% ophthalmic emulsion (Ocurin, Bilim Pharmaceuticals, İstanbul, Türkiye) was prescribed as an adjunctive therapy to the conservative treatment protocol. The cyclosporine drops were administered twice daily.

#### 2.8.5. Group 5 (Conservative Management with Adjunctive IPL Therapy)

The IPL protocol was implemented, together with conservative management, on days 0, 15, and 30.

### 2.9. Statistical Analyses

Statistical analysis was performed using SPSS version 27 software. A priori power analysis using G Power 3.1.9.7 (1 − β = 0.80, α = 0.05, f = 0.40) indicated a minimum requirement of 16 participants per group. The conformity of variables with a normal distribution was examined using the Kolmogorov–Smirnov/Shapiro–Wilk tests. Descriptive statistics were expressed as the mean ± standard deviation (SD) or median values for continuous data and as a number (n) and percentage (%) for categorical data. The Wilcoxon signed-rank test was used to compare pre- and post-treatment values within each group. The Kruskal–Wallis H test was applied to compare changes in values between groups. A value of *p* < 0.05 was considered statistically significant.

## 3. Results

The 92 subjects enrolled in this study consisted of 50 (64.2%) women and 40 (35.8%) men, with a mean age of 45.16 ± 7.28 years.

Group 1 consisted of eighteen members (six males, twelve females), with a mean age of 46.67 years. Group 2 (doxycycline) contained twenty-two members (eight males, fourteen females), with a mean age of 45.38 years. Group 3 (azithromycin) consisted of eighteen members (six males, twelve females), with a mean age of 46.44 years. Group 4 (cyclosporine) contained eighteen members (five males, thirteen females), with a mean age of 47.11 years, and Group 5 (IPL) contained sixteen members (five males, eleven females), with a mean age of 43.88 years. No statistically significant differences were observed in age or gender distributions among the treatment groups (*p* > 0.05).

### 3.1. Group 1 (Conservative Treatment Only)

Group 1: TBUT values exhibited a statistically insignificant change from 9.00 ± 2.67 to 9.94 ± 2.41 s (*p* > 0.05), while lid vascularity scores decreased significantly from 1.67 ± 0.84 to 1.06 ± 0.8 (*p* = 0.005). Meibomian gland plugging exhibited no statistically significant change, decreasing from 1.11 ± 0.58 to 0.94 ± 0.42 (*p* = 0.083). Lid margin irregularity decreased significantly from 0.67 ± 0.49 to 0.33 ± 0.49 (*p* = 0.014). Foaming scores exhibited a statistically insignificant change from 0.44 ± 0.51 to 0.89 ± 1.57 (*p* = 0.157). Marx line scores exhibited significant improvement from 4.28 ± 0.89 to 3.33 ± 1.19 (*p* = 0.003). Meibum grade exhibited a statistically insignificant change from 1.67 ± 0.49 to 1.50 ± 0.51 (*p* = 0.083). Patient-reported outcomes improved significantly, with OSDI scores decreasing from 51.67 ± 7.55 to 39.89 ± 8.83 (*p* < 0.001) and SPEED scores from 13.00 ± 4.39 to 9.94 ± 2.41 (*p* = 0.01).

### 3.2. Group 2 (Doxycycline)

Group 2: TBUT values increased significantly from 8.15 ± 3.23 to 10.58 ± 3.11 s (*p* = 0.027). A statistically significant decrease was determined in lid vascularity values, from 2.08 ± 0.93 to 1.46 ± 0.71 (*p* = 0.008), and in meibomian gland plugging values, from 1.54 ± 0.76 to 1.12 ± 0.43 (*p* < 0.001). Lid margin irregularity exhibited a statistically insignificant change from 0.85 ± 0.54 to 0.58 ± 0.5 (*p* = 0.07), foaming displayed an insignificant change from 0.85 ± 0.97 to 0.58 ± 0.76 (*p* = 0.07), and Marx line scores showed a significant improvement from 5.46 ± 1.75 to 4.62 ± 1.1 (*p* = 0.005). Statistically significant improvements were also observed in meibum grades, from 2.38 ± 0.5 to 1.54 ± 0.58 (*p* < 0.001), in OSDI, from 46.77 ± 16.22 to 33.77 ± 8.37 (*p* < 0.001), and in SPEED, from 15.46 ± 4.19 to 10.58 ± 3.11 (*p* < 0.001).

### 3.3. Group 3 (Azithromycin)

Group 3: TBUT exhibited a statistically insignificant change from 6.83 ± 1.24 to 9.00 ± 1.28 s (*p* = 0.109). Lid vascularity decreased significantly from 2.00 ± 0.97 to 1.33 ± 0.69 (*p* = 0.007), and meibomian gland plugging decreased significantly from 1.44 ± 0.7 to 1.00 ± 0 (*p* = 0.023). Lid margin irregularity improved significantly from 1.11 ± 0.32 to 0.89 ± 0.58 (*p* = 0.046), while foaming changed insignificantly from 0.89 ± 0.58 to 0.56 ± 0.51 (*p* = 0.063), and Marx line scores changed insignificantly from 5.89 ± 1.45 to 5.50 ± 1.65 (*p* = 0.071). Meibum grade improved significantly from 1.89 ± 0.58 to 1.44 ± 0.51 (*p* = 0.005). Statistically significant improvements were observed in patient-reported outcomes, with a decrease in OSDI from 41.11 ± 18.7 to 27.33 ± 7.91 (*p* = 0.003) and in SPEED from 13.44 ± 6.02 to 9.00 ± 1.28 (*p* = 0.01).

### 3.4. Group 4 (Cyclosporine)

Group 4: TBUT increased significantly from 7.11 ± 1.32 to 9.17 ± 0.98 s (*p* = 0.007). Lid vascularity exhibited significant improvement, from 1.78 ± 0.94 to 1.28 ± 0.75 (*p* = 0.014). Meibomian gland plugging decreased significantly from 1.11 ± 0.32 to 0.89 ± 0.58 (*p* = 0.046), while lid margin irregularity exhibited no statistically significant change, increasing from 0.33 ± 0.49 to 0.44 ± 0.51 (*p* = 0.157), and foaming scores remained stable at 0.33 ± 0.49 (*p* = 1.000). Marx line scores exhibited no statistically significant change, decreasing from 4.00 ± 0.84 to 3.89 ± 1.28 (*p* = 0.729). A significant improvement was observed in the meibum grade, from 1.78 ± 0.65 to 1.44 ± 0.7 (*p* = 0.014). Patient-reported outcomes exhibited significant improvements, with decreases in OSDI from 52.33 ± 14.66 to 40.33 ± 9.44 (*p* < 0.001) and in SPEED from 15.22 ± 4.39 to 9.17 ± 0.99 (*p* < 0.001).

### 3.5. Group 5 (IPL)

Group 5: TBUT exhibited a statistically insignificant change from 7.20 ± 1.13 to 10.40 ± 0.51 s (*p* = 0.066). Lid vascularity scores decreased significantly from 1.80 ± 1.03 to 1.00 ± 0 (*p* = 0.046), and meibomian gland plugging decreased significantly from 1.40 ± 0.52 to 1.00 ± 0.94 (*p* = 0.046). Pre- and post-treatment lid margin irregularity remained stable at 0.80 ± 0.79 (*p* = 1.000), and foaming scores were maintained at 0.20 ± 0.42 (*p* = 1.000). A statistically significant improvement was observed in Marx line scores, declining from 4.80 ± 1.23 to 3.60 ± 0.52 (*p* = 0.014), and in meibum grades, decreasing from 2.00 ± 0.67 to 1.60 ± 0.84 (*p* = 0.046). Patient-reported outcomes also improved significantly, with decreases in OSDI scores from 47.20 ± 20.06 to 19.60 ± 7.17 (*p* = 0.005), and from 17.60 ± 3.86 to 10.40 ± 0.52 in SPEED (*p* = 0.004) (Table 2).

TBUT values increased significantly in Group 2, and lid vascularity scores decreased significantly in all groups. Lid margin irregularity decreased significantly in Group 1. Meibomian gland plugging decreased significantly in Groups 2, 3, 4, and 5. Marx line scores exhibited a significant improvement in Groups 1, 2, and 5. Meibum grades decreased significantly in Groups 2, 3, 4, and 5. OSDI and SPEED scores improved significantly in all groups (Table 3).

### 3.6. Analysis of Treatment Efficacy

The meibum grade exhibited the most pronounced treatment-related differences (*p* = 0.009). Group 2 registered the highest improvement, with a mean percentage change of 35.26 ± 21.77%, significantly outperforming the group receiving conservative treatment alone (8.33 ± 19.17%). The Kruskal–Wallis H analysis revealed significant pairwise differences between Group 2 and Group 1 (*p* < 0.001), and between Group 2 and Group 4 (*p* = 0.005). Groups 3, 4, and 5 registered moderate improvements of 20.37%, 16.67%, and 20%, respectively. OSDI scores exhibited significant inter-group variation (*p* = 0.015), with Group 5 (IPL therapy) registering markedly elevated scores (54.06 ± 21.93%) compared to all other treatment modalities. The Kruskal–Wallis H analysis demonstrated significant differences between Group 5 and conservative treatment alone (*p* < 0.001), Group 2 (*p* < 0.001), Group 3 (*p* < 0.001), and Group 4 (*p* < 0.001). Groups 1–4 exhibited relatively similar OSDI improvements ranging from 20.87% to 23.36%. The foaming assessment revealed significant treatment effects (*p* = 0.018). Notably, Group 1 registered negative values (−100 ± 185.16%), indicating potential deterioration. Group 2 achieved the highest positive response (40.48 ± 47.46%), with significant pairwise differences compared to Group 1 (*p* < 0.001) and Group 3 (*p* = 0.002). Groups 4 and 5 exhibited no measurable foaming changes (0 ± 0%). Several clinical parameters, including lid vascularity (*p* = 0.540), plugging (*p* = 0.521), lid margin irregularity (*p* = 0.081), Marx line score (*p* = 0.075), Schirmer test speed (*p* = 0.471), and TBUT (*p* = 0.124), exhibited no statistically significant inter-group differences, suggesting that these parameters may be less responsive to the evaluated treatment modalities or else require longer observation periods to detect meaningful changes (Table 4).

The Wilcoxon signed-rank test was used to compare pre- and post-treatment values within each group. *p* < 0.05 is regarded as statistically significant.

## 4. Discussion

Meibomian gland dysfunction is an increasingly prevalent ocular surface disorder characterized by terminal duct obstruction and/or qualitative/quantitative changes in glandular secretion. This results in altered tear film lipid layers, evaporative dry eye, and chronic ocular surface inflammation. The multifactorial nature of MGD calls for equally diverse therapeutic approaches [24]. While several studies have compared oral treatment modalities for MGD, no comparative research has specifically evaluated the effectiveness of cyclosporine versus IPL therapy.

Conservative management (preservative-free artificial tears, warm compresses, eyelid massage, and eyelash cleansing) constitutes the basis of MGD treatment, providing relief for mild cases by improving meibum properties and tear film stability. However, this approach alone often fails to address underlying glandular abnormalities in moderate-to-severe cases or those with persistent inflammation, necessitating adjunctive therapies to target the complex pathophysiology of MGD.

Adjunctive oral antibiotic therapy was evaluated in Groups 2 and 3 in this study. Doxycycline, a tetracycline antibiotic, exerts anti-inflammatory effects by inhibiting matrix metalloproteinases and modifying the lipid composition of meibum, which together reduce inflammation and enhance glandular function. In contrast, oral azithromycin provides the advantage of a shorter treatment course, improved patient compliance, and a lower incidence of adverse effects. The clinical distinction between these antibiotics is significant, since effective adjunctive antibiotic therapy in MGD hinges on both antimicrobial activity and the modulation of inflammatory pathways. The current findings support previous studies reporting that doxycycline treatment led to improved TBUT, while both treatments yielded reduced meibomian gland plugging and better subjective patient outcomes. A greater improvement in eyelid symptoms was also observed with azithromycin than with doxycycline. Kashkouli et al. and Bukhari et al. compared oral azithromycin and doxycycline and reported that azithromycin was more effective against conjunctival redness and corneal staining [25,26,27]. In another study comparing topical azithromycin and oral doxycycline therapy, oral doxycycline administration caused a greater prolongation of tear break-up time, whereas topical azithromycin had a greater effect on symptoms [11].

Topical cyclosporine was incorporated in the treatment of Group 4 in this study, reflecting an alternative therapeutic approach that leverages immunomodulation. Topical cyclosporine is known to suppress T-cell activity and reduce ocular surface inflammation [6,28]. Although primarily used in the management of chronic dry eye syndrome, its role in MGD is gaining increasing recognition, particularly in cases in which inflammation is a dominant feature. The statistically significant improvement in lid vascularity and meibum quality observed in this group suggests that localized immunomodulatory therapy may have the potential for integration into a comprehensive treatment regimen for MGD. These preliminary findings suggest a potential alternative option, especially for patients who might be contraindicated for systemic therapy or who experience adverse effects related to systemic antibiotics. Jeon et al. reported that patients treated with a combination of cyclosporine and IPL exhibited decreased inflammatory markers and improved eyelid condition. These results hint at a possible synergistic effect between the two treatments, tackling inflammation and enhancing clinical signs in a complementary manner [29]. Iaccheri et al. applied artificial tear drop therapy alone to one group of patients with MGD-related dry eye and artificial tear drop therapy together with cyclosporine drops to the other group. Those authors observed a significant improvement in meibum expression and quality from the first months in the group in which cyclosporine treatment was added [30].

Group 5 in the current study, who received adjunctive IPL therapy, registered significant clinical improvements at one month post-treatment. The thermal effects of IPL appear to liquefy meibum and reduce abnormal telangiectatic vessels, thereby modulating inflammatory mediators and enhancing the ocular surface microenvironment. In this study, IPL therapy was associated with decreased meibomian gland plugging and improved Marx line scores and meibum grade. These results align with previous findings that indicate the efficacy of IPL in reducing MGD symptoms and improving both eyelid and gland functions [31].

The differential treatment responses observed across the evaluated parameters provide important insights into the pathophysiological mechanisms underlying MGD and the varying therapeutic approaches investigated. The notable improvements observed in Group 2 in improving meibum grade, with a 35.26% improvement rate representing a substantial improvement compared to baseline over Group 1, highlight the critical role of systemic anti-inflammatory intervention in addressing the underlying inflammatory cascade characteristic of MGD. This finding aligns with the existing understanding of doxycycline’s dual mechanism of action, encompassing both antimicrobial properties and potent anti-inflammatory effects through matrix metalloproteinase inhibition and cytokine modulation. The significant superiority of Group 2 over Group 4 in short-term outcomes suggests that systemic approaches may be more effective in rapidly addressing the multifactorial inflammatory processes involved in meibomian gland dysfunction. The paradoxical 54.06% elevation in OSDI scores observed in Group 5 warrants careful consideration and may reflect several underlying mechanisms. This finding may indicate an initial inflammatory response to IPL, suggesting that patients may experience temporary symptom exacerbation before achieving therapeutic benefit. Alternatively, it may suggest that the observation period was insufficient to capture the full therapeutic response of this modality. The consistent OSDI improvement rates across the other four treatment groups (Groups 1, 2, 3, and 4, ranging from 20% to 23%) demonstrate the reliability of conventional therapeutic approaches in providing predictable symptomatic relief in the short term. The foaming parameter assessment revealed particularly informative patterns, with the negative values (−100%) in Group 1 highlighting the progressive nature of untreated MGD and emphasizing the importance of active intervention. The substantial positive response (40.48%) in this parameter in Group 2 further confirms its restorative effects on meibomian gland function, while the absence of measurable changes in Groups 4 and 5 suggests either distinct mechanisms of action or the need for extended treatment periods to demonstrate efficacy in this specific functional parameter. The lack of significant inter-group differences in traditional parameters such as the Schirmer test and TBUT indicates that these conventional measures may possess limited sensitivity for detecting short-term treatment responses, highlighting the importance of incorporating more specific functional assessments, such as meibum quality and foaming characteristics, in the evaluation of MGD therapeutic interventions.

While conservative management constitutes the basis of MGD treatment, adjunctive therapies demonstrated distinct response patterns compared to conservative management alone. Systemic antibiotics and topical cyclosporine were determined to improve tear film stability and reduce inflammation, whereas IPL therapy demonstrated distinct response patterns in comprehensive management, exhibiting notable improvements in both obstructive and inflammatory parameters.

While the absence of a placebo control group represents a potential limitation of this research, our study design incorporates several robust methodological features that mitigate this concern. The parallel evaluation of five mechanistically distinct treatment modalities provides compelling evidence that the observed outcomes extend beyond mere placebo effects. Had improvement been driven solely by patient expectancy, uniform therapeutic gains would be anticipated across all treatment arms; however, our findings demonstrate distinct efficacy profiles characteristic of each individual treatment modality. The IPL arm registered the most notable increase in TBUT (+3.20 s) and a substantial decrease in lid margin scores (−1.20), while the purely conservative warm-compress and massage arm exhibited the smallest change (+0.94 s; −0.34). The study findings demonstrated consistent treatment responses across semi-objective measures (TBUT, lid margin grading, and meibum expressibility), with OSDI scores improving by more than the eight-point minimal clinically important difference in all treatment arms (−12.8 to −27.6 points). Nevertheless, the methodological constraints inherent to semi-objective assessments also warrant consideration. Semi-objective clinical assessments may introduce ±15–20% measurement variability compared to automated diagnostic systems, while TBUT evaluations potentially exhibit 10–15% inter-observer discrepancy despite standardization efforts. The reliance on semi-objective clinical parameters and patient-reported questionnaires means that these measures may not fully capture the underlying pathophysiological changes in MGD. However, the substantial treatment effects observed exceed typical measurement variability, supporting the validity of our findings.

Several important considerations emerge when interpreting the generalizability of our findings across different populations and clinical settings. The relatively homogeneous age distribution in the study population provides valuable insights into treatment efficacy within this demographic. In addition, the well-established age-related variations in MGD severity and treatment response suggest that therapeutic outcomes may differ across younger or geriatric populations. Meibomian gland morphology, lipid composition, and inflammatory responses undergo significant changes with aging, potentially affecting therapeutic efficacy across different age demographics. Furthermore, our study demonstrates robust treatment efficacy within a specialized ophthalmology clinic population, though the translation of these findings to broader community-based MGD patients requires careful consideration. The controlled clinical environment, specialized diagnostic capabilities, and potentially higher treatment adherence rates characteristic of our study setting may have contributed to optimized therapeutic outcomes that may differ from those in routine practice settings. These considerations highlight important opportunities for future research. Subsequent investigations would benefit from incorporating age-stratified analytical approaches and community-based recruitment strategies in order to further enhance our understanding of MGD treatment efficacy. Such studies would better elucidate differential treatment responses across distinct age demographics and diverse clinical settings, particularly considering the complex interplay between age-related alterations in meibomian gland architecture, lipid biochemistry, inflammatory cascades, and real-world treatment implementation.

### Limitations

The primary limitation of this study is the small sample size in each treatment group, which limits statistical power for definitive comparative conclusions. With multiple clinical parameters across five treatment modalities, this descriptive pilot study characterizes treatment response patterns rather than providing definitive comparative assessments. Larger sample sizes will be required for robust comparative analyses in future studies.

Since topical azithromycin is not available in Türkiye, this could not be included in this research, and no comparison could therefore be made with oral azithromycin. Another significant limitation is that the one-month follow-up period, which, while appropriate for characterizing early treatment response patterns in this pilot study, may not have been sufficient to fully capture the long-term therapeutic benefits of treatments such as topical cyclosporine, which demonstrates progressive improvement with extended use. Future studies should incorporate longer follow-up periods to comprehensively evaluate sustained therapeutic effects. Additionally, this study did not employ age stratification, which may facilitate a better assessment of differential treatment responses across age groups in future MGD treatment studies. Variations in follow-up periods in the literature also pose challenges in terms of a direct comparison between the current findings and those of previous studies. In the present study, OSDI and SPEED questionnaire tests were applied to evaluate patient-reported symptoms. The scores improved in all patients. Questionnaire tests are a limiting factor because they yield subjective information and can be affected by environmental factors. Additionally, the absence of a placebo control group limits our ability to distinguish genuine treatment effects from potential placebo responses, particularly for subjective outcome measures. Moreover, the absence of fully device-based objective metrics such as tear film osmolarity testing or infrared meibography meant that outcome assessment relied on semi-objective clinical measures, thereby constraining the absolute objectivity of the study results. Finally, our study population was recruited from a single ophthalmology clinic, and this may introduce selection bias and limit generalizability, since these patients may differ systematically from community-based MGD populations in terms of health literacy, trust in medical interventions, symptom awareness, and healthcare-seeking behaviors.

## 5. Conclusions

This descriptive pilot study characterized the clinical evolution patterns of five therapeutic approaches toward MGD management. Conservative management provided baseline therapeutic effects, while adjunctive therapies (doxycycline, cyclosporine, and IPL) exhibited distinct response patterns in different clinical parameters. Each treatment modality exhibited unique characteristics in terms of parameter improvements and patient-reported outcomes. These preliminary findings suggest potential for personalized, multimodal approaches toward MGD management, with treatment selection guided by individual patient characteristics. The comprehensive evaluation of MGD treatment requires extended follow-up periods in order to fully capture the therapeutic evolution required for chronic disease management, particularly for treatments demonstrating progressive, time-dependent improvement. In order to maximize the clinical value of these extended assessments, future investigations should integrate placebo-controlled designs with comprehensive objective diagnostic modalities, including tear film osmolarity testing, infrared meibography, and inflammatory biomarker analysis. This will enhance measurement precision, control for placebo bias, and improve diagnostic accuracy throughout the longitudinal evaluation process.

## Figures and Tables

**Table 1:** Grading scales for the evaluation criteria of meibomian gland dysfunction [14,19,20,21].

Lid Vascularity	
0	None
1	Redness of the palpebral conjunctiva with no vascularity around the gland orifices
2	Redness of the palpebral conjunctiva with vascularity around the gland orifices affecting <50% of the full length of the lid margin
3	Redness of the palpebral conjunctiva with vascularity around the gland orifices affecting ≥50% of the full length of the lid margin
**Lid margin irregularity**	
0	None
1	Fewer than three lid margin irregularities with shallow notching
2	Three or more lid margin irregularities or deep notching
**Foaming**	
0	None
1	Mild findings
2	Severe findings
**Lid plugging**	
0	None
1	Plugging of <3 gland orifices
2	Plugging of ≥3 gland orifices affecting <50% of the full length of the lid margin
3	Plugging of ≥3 gland orifices affecting ≥50% of the full length of the lid margin
**Marx line score**	
0	Marx line running entirely along the conjunctival side of the gland orifices
1	Part of the Marx line in contact with the gland orifices
2	Marx line running through the gland orifices
3	Marx line running along the eyelid margin on the side of the gland orifices
**Meibum grade**	
0	Clear meibum is easily expressed
1	Cloudy meibum is expressed with mild pressure
2	Cloudy meibum is expressed with more than moderate pressure
3	Meibum cannot be expressed even with the hard pressure

**Table 2:** Comparisons of pre- and post-treatment clinical parameters in patients with meibomian gland dysfunction across the different treatments.

	Group 1—Conservative Treatment Only		Group 2—Conservative Treatment + Oral Doxycycline		Group 3—Conservative Treatment + Oral Azithromycin		Group 4—Conservative Treatment + Cyclosporine		Group 5—Conservative Treatment + IPL Therapy	
Clinical Parameters	Pre-treatment (Mean ± SD)	Post-treatment (Mean ± SD)	Pre-treatment (Mean ± SD)	Post-treatment (Mean ± SD)	Pre-treatment (Mean ± SD)	Post-treatment (Mean ± SD)	Pre-treatment (Mean ± SD)	Post-treatment (Mean ± SD)	Pre-treatment (Mean ± SD)	Post-treatment (Mean ± SD)
Lid vascularity (score) (0–3)	1.67 ± 0.84	1.06 ± 0.8	2.08 ± 0.93	1.46 ± 0.71	2 ± 0.97	1.33 ± 0.69	1.78 ± 0.94	1.28 ± 0.75	1.8 ± 1.03	1 ± 0
Lid Plugging	1.11 ± 0.58	0.94 ± 0.42	1.54 ± 0.76	1.12 ± 0.43	1.44 ± 0.7	1 ± 0	1.11 ± 0.32	0.89 ± 0.58	1.4 ± 0.52	1 ± 0.94
Lid margin irregularity (score) (0–2)	0.67 ± 0.49	0.33 ± 0.49	0.85 ± 0.54	0.58 ± 0.5	1.11 ± 0.32	0.89 ± 0.58	0.33 ± 0.49	0.44 ± 0.51	0.8 ± 0.79	0.8 ± 0.79
Foaming (score) (0–2)	0.44 ± 0.51	0.89 ± 1.57	0.85 ± 0.97	0.58 ± 0.76	0.89 ± 0.58	0.56 ± 0.51	0.33 ± 0.49	0.33 ± 0.49	0.2 ± 0.42	0.2 ± 0.42
Marx line (score) (0–3)	4.28 ± 0.89	3.33 ± 1.19	5.46 ± 1.75	4.62 ± 1.1	5.89 ± 1.45	5.5 ± 1.65	4 ± 0.84	3.89 ± 1.28	4.8 ± 1.23	3.6 ± 0.52
Meibum grade (Score) (0–3)	1.67 ± 0.49	1.5 ± 0.51	2.38 ± 0.5	1.54 ± 0.58	1.89 ± 0.58	1.44 ± 0.51	1.78 ± 0.65	1.44 ± 0.7	2 ± 0.67	1.6 ± 0.84
OSDI (score) (0–100)	51.67 ± 7.55	39.89 ± 8.83	46.77 ± 16.22	33.77 ± 8.37	41.11 ± 18.7	27.33 ± 7.91	52.33 ± 14.66	40.33 ± 9.44	47.2 ± 20.06	19.6 ± 7.17
Speed (Score) (0–28)	13 ± 4.39	9.94 ± 2.41	15.46 ± 4.19	10.58 ± 3.11	13.44 ± 6.02	9 ± 1.28	15.22 ± 4.39	9.17 ± 0.99	17.6 ± 3.86	10.4 ± 0.52
TBUT (seconds)	9.00 ± 2.67	9.94 ± 2.41	8.15 ± 3.23	10.58 ± 3.11	6.83 ± 1.24	9.00 ± 1.28	7.11 ± 1.32	9.17 ± 0.98	7.20 ± 1.13	10.40 ± 0.51

**Table 3:** Significance values of changes before and after treatment in the study groups.

Clinical Parameters	*p*-Values for Group 1	*p*-Values for Group 2	*p*-Values for Group 3	*p*-Values for Group 4	*p*-Values for Group 5
Lid vascularity	**0.005**	**0.008**	**0.007**	**0.014**	**0.046**
Lid Plugging	0.083	**<0.001**	**0.023**	**0.046**	**0.046**
Lid margin irregularity	**0.014**	0.07	**0.046**	0.157	1.000
Foaming	0.157	0.07	0.063	1.000	1.000
Marx line score	**0.003**	**0.005**	0.071	0.729	**0.014**
Meibum grade	0.083	**<0.001**	**0.005**	**0.014**	**0.046**
OSDI	**<0.001**	**<0.001**	**0.003**	**<0.001**	**0.005**
Speed	**0.01**	**<0.001**	**0.01**	**<0.001**	**0.004**
TBUT	0.685	**0.027**	0.109	**0.007**	0.066

***p* < 0.05** was considered statistically significant.

**Table 4:** A comparison of percentage changes in clinical parameters relative to the baseline among the meibomian gland dysfunction treatment groups.

Clinical Parameters	Group 1—Conservative Treatment Only (a)	Group 2—Conservative Treatment + Oral Doxycycline (b)	Group 3—Conservative Treatment + Oral Azithromycin (c)	Group 4—Conservative Treatment + Cyclosporine (d)	Group 5—Conservative Treatment + IPL Therapy (e)	*p*
Lid vascularity	(38.89) ± (44.65)	(16.67) ± (50.77)	(37.5) ± (23.96)	(22.22) ± (31.83)	(26.67) ± (34.43)	0.540
Lid Plugging	(9.38) ± (20.16)	(20.51) ± (27.21)	(18.52) ± (27.35)	(22.22) ± (42.78)	(40) ± (51.64)	0.521
Lid margin irregularity	(50) ± (52.22)	(35) ± (48.94)	(22.22) ± (42.78)	(0) ± (0)	(0) ± (0)	0.081
Foaming	(−100) ± (185.16)	(40.48) ± (47.46)	(28.57) ± (46.88)	(0) ± (0)	(0) ± (0)	**0.018** ***p* ^ab^ < 0.001** ***p* ^ac^ = 0.002**
Marx line score	(22.13) ± (23.71)	(11.86) ± (22.03)	(6.79) ± (15.96)	(3.15) ± (24.12)	(21.33) ± (18.54)	0.075
Meibum grade	(8.33) ± (19.17)	(35.26) ± (21.77)	(20.37) ± (23.95)	(16.67) ± (24.25)	(20) ± (25.82)	**0.009** ***p* ^ab^ < 0.001** ***p* ^bd^ = 0.005**
OSDI	(23.14) ± (10.9)	(23.36) ± (18.4)	(22.86) ± (29.13)	(20.87) ± (12.02)	(54.06) ± (21.93)	**0.015** ***p* ^ae^ < 0.001** ***p* ^be^ < 0.001** ***p* ^ce^ < 0.001** ***p* ^de^ < 0.001**
Speed	(12.34) ± (40.2)	(26.56) ± (27.26)	(11.46) ± (63.78)	(33.97) ± (24.6)	(38.93) ± (10.13)	0.471
TBUT	(−0.92) ± (47.13)	(18.1) ± (52.71)	(41.68) ± (76.03)	(57.59) ± (76.61)	(41.14) ± (55.55)	0.124

Parameters exhibiting statistically significant differences were subjected to pairwise comparisons using the Kruskal–Wallis H test. A ***p* < 0.05** was considered statistically significant. a: Group 1—Conservative Treatment Only, b: Group 2—Conservative Treatment + Oral Doxycycline, c: Group 3—Conservative Treatment + Oral Azithromycin, d: Group 4—Conservative Treatment + Cyclosporine, e: Group 5—Conservative Treatment + IPL Therapy.

## Data Availability

Data supporting the findings of this study can be obtained from the corresponding author upon reasonable request. The dataset is not publicly available owing to ethical and confidentiality constraints.

## Supplementary Materials

The following supporting information can be downloaded at: https://www.mdpi.com/article/10.3390/healthcare13161992/s1, Supplementary Table S1 presents the OSDI questionnaire items with the scoring rubric and the computation formula for the total score. Supplementary Table S2 provides the SPEED questionnaire items and response options for frequency and severity, suitable for replication and standardized data collection.

## Abbreviations

Meibomian gland dysfunction (MGD), intense pulsed light (IPL) therapy, Ocular Surface Disease Index (OSDI), tear break-up time (TBUT), Standard Patient Evaluation of Eye Dryness (SPEED), meibomian orifices (MOs).
