# Supplementary material for: A Short-Term Comparative Evaluation of Multiple Treatment Modalities for Meibomian Gland Dysfunction: A Prospective Clinical Study
Source: Healthcare (Basel). 2025 Aug 14;13(16):1992. doi: 10.3390/healthcare13161992 (PMC12385541; doi:10.3390/healthcare13161992)
Supplement: Supplementary file 1 [file healthcare-13-01992-s001.zip › healthcare-3770861-supplementary.pdf]

Supplementary Table S1: OSDI Questionnaire (Ocular Surface Disease Index) (Items and Response Scale)

1. **Have you experienced any of the following during the last week:**						
	All of the time	Most of the time	Half of the time	Some of the time	None of the time	
1. Eyes that are sensitive to light	4	3	2	1	0	
2. Eyes that feel gritty?	4	3	2	1	0	
3. Painful or sore eyes?	4	3	2	1	0	
4. Blurred vision?	4	3	2	1	0	
5. Poor vision?	4	3	2	1	0	
2. **Have problems with your eyes limited you in performing any of the following during the last week:**						
	All of the time	Most of the time	Half of the time	Some of the time	None of the time	
6. Reading?	4	3	2	1	0	N/A
7. Driving at night?	4	3	2	1	0	N/A
8. Working with a computer or a bank	4	3	2	1	0	N/A

machine(ATM)?						
9. Watching TV?	4	3	2	1	0	N/A
**3. Have your eyes felt unfortable in any of the following situations during the last week:**						
	All of the time	Most of the time	Half of the time	Some of the time	None of the time	
10. Windy conditions?	4	3	2	1	0	N/A
11. Places or areas with low humidity(very dry)?	4	3	2	1	0	N/A
12. Areas that are air conditioned?	4	3	2	1	0	N/A

Formula: OSDI = (sum of answered item scores × 25) / number of answered items.

Supplementary Table S2: SPEED™ Questionnaire (Standardized Patient Evaluation of Eye Dryness [SPEED] Questionnaire)

1. Report the type of SYMPTOMS you experience and when they occur:

Symptoms	At this visit	Within past 72 hours	Within past 3 months
	Yes	No	Yes
Dryness, Grittiness or Scratchiness			
Soreness or Irritation			
Burning or Watering			
Eye Fatigue			

2. Report the FREQUENCY of your symptoms using the rating list below:

Symptoms	0	1	2	3
Dryness, Grittiness or Scratchiness				
Soreness or Irritation				
Burning or Watering				
Eye Fatigue				

0 = Never 1 = Sometimes 2 = Often 3 = Constant

3. Report the SEVERITY of your symptoms using the rating list below:

Symptoms	0	1	2	3	4
Dryness, Grittiness or Scratchiness					
Soreness or Irritation					
Burning or Watering					
Eye Fatigue					

0 = No Problems 1 = Tolerable - not perfect, but not uncomfortable 2 = Uncomfortable - irritating, but does not interfere with my day 3 = Bothersome - irritating and interferes with my day 4 = Intolerable - unable to perform my daily tasks

4. Do you use eye drops for lubrication? □ YES □ NO If yes, how often? _______________

For office use only Total SPEED score (Frequency + Severity) = ___
